# Adequate Iodine Status in New Zealand School Children Post-Fortification of Bread with Iodised Salt

**DOI:** 10.3390/nu8050298

**Published:** 2016-05-16

**Authors:** Emma Jones, Rachael McLean, Briar Davies, Rochelle Hawkins, Eva Meiklejohn, Zheng Feei Ma, Sheila Skeaff

**Affiliations:** 1 Department of Human Nutrition, University of Otago, Dunedin 9054, New Zealand; emmajones.nz@gmail.com (E.J.); rachael.mclean@otago.ac.nz (R.M.); briardavies@gmail.com (B.D.); rkhawkins039@gmail.com (R.H.); evameiklejohn@gmail.com (E.M.); zhengfeei.ma@postgrad.otago.ac.nz (Z.F.M.); 2 Department of Preventive and Social Medicine, University of Otago, Dunedin 9054, New Zealand

**Keywords:** iodine, iodised salt, fortification, deficiency, children, New Zealand

## Abstract

Iodine deficiency re-emerged in New Zealand in the 1990s, prompting the mandatory fortification of bread with iodised salt from 2009. This study aimed to determine the iodine status of New Zealand children when the fortification of bread was well established. A cross-sectional survey of children aged 8–10 years was conducted in the cities of Auckland and Christchurch, New Zealand, from March to May 2015. Children provided a spot urine sample for the determination of urinary iodine concentration (UIC), a fingerpick blood sample for Thyroglobulin (Tg) concentration, and completed a questionnaire ascertaining socio-demographic information that also included an iodine-specific food frequency questionnaire (FFQ). The FFQ was used to estimate iodine intake from all main food sources including bread and iodised salt. The median UIC for all children (*n* = 415) was 116 μg/L (females 106 μg/L, males 131 μg/L) indicative of adequate iodine status according to the World Health Organisation (WHO, *i.e.*, median UIC of 100–199 μg/L). The median Tg concentration was 8.7 μg/L, which was <10 μg/L confirming adequate iodine status. There was a significant difference in UIC by sex (*p* = 0.001) and ethnicity (*p* = 0.006). The mean iodine intake from the food-only model was 65 μg/day. Bread contributed 51% of total iodine intake in the food-only model, providing a mean iodine intake of 35 μg/day. The mean iodine intake from the food-plus-iodised salt model was 101 μg/day. In conclusion, the results of this study confirm that the iodine status in New Zealand school children is now adequate.

## 1. Introduction

New Zealand has a history of iodine deficiency due to the naturally low levels of iodine in the soil. Studies conducted in the 1920s found that a large proportion of school-aged children had goiter [1,2]. This finding prompted a national public health initiative to improve iodine intakes, and in 1924 iodised salt was introduced at a level of 5 ppm [3]. This level of iodisation proved to be inadequate as iodine deficiency still persisted into the 1930s, prompting an increase in iodisation to 40–80 ppm in 1938. As a result, by the early 1950s iodine status in New Zealand had improved and goitre virtually disappeared [3]. Iodine intakes further increased in the 1960s when the dairy industry began using iodophors as a sanitizer, which contaminated milk thereby increasing the iodine content of dairy products. However, the replacement of iodophors in the dairy industry together with a change in consumer food habits such as a decrease in the use of discretionary salt and the rising popularity of non-iodised rock and sea salts, led to a decline in iodine intake [3]. In response to several studies confirming mild iodine deficiency among children and adults since the 1990s [4,5,6,7], Food Standards Australia New Zealand (FSANZ) mandated the use of iodised salt in bread from 2009. Standard 2.1.1 requires that “iodised salt be used for making bread where salt would otherwise be used” where bread is defined as “the product made by baking a yeast-leavened dough prepared from one or more cereal flours or meals and water”. This includes products made from bread dough containing yeast and salt such as bread loaves, buns and rolls, wholemeal and multigrain, flat breads, bagels, English muffins and sweet buns. However, this requirement does not apply to bread represented as organic, the addition of salt to the surface of bread (e.g., rock salt), or the addition of other ingredients containing salt during the making of bread [8].

Mandatory fortification of bread with iodised salt has improved the iodine status of Australian children. The median UIC of 8–10 years old Australian children (*n* = 1709) prior to fortification was 96 μg/L [9]; the World Health Organisation (WHO) states that a median UIC of a group or population between 50 and 100 μg/L indicates mild deficiency [10]. Post-fortification, the 2011/13 Australian National Health Survey [11] sampled over 11,000 children aged 5–11 years and reported a median UIC of 176 μg/L, categorizing Australian children as having adequate iodine status. To date, only one small study has evaluated the effect of the mandatory fortification of bread on the iodine status of New Zealand children. In 2011, Skeaff and Lonsdale-Cooper [12] found that 8–10 years old children (*n* = 147) living in Wellington and Dunedin had a median UIC of 113 μg/L, however, the median Tg concentration of the children was 12.9 μg/L, above the recommended cut-off of 10 μg/L [10]. Tg is an indirect index of thyroid volume (*i.e.*, goiter) and increases when thyroid volume is higher than normal [13], leading the authors to speculate that insufficient time (<two years) had passed since fortification for goiter to resolve. The aim of the current study was to extend the 2011 study by recruiting a more representative sample of children from the two other main urban centers in New Zealand with a longer interval between the introduction of fortified bread and the assessment of iodine status.

## 2. Materials and Methods

### 2.1. Study Design and Recruitment

A cross-sectional study was conducted from March to April 2015 in two New Zealand cities: Christchurch in the South Island and Auckland in the North Island. Children were included in the study if they were aged between 8 and 10 years and had not been medically diagnosed with thyroid disease. In brief, iodine status was determined by measuring the urinary iodine concentration (UIC) in a spot urine sample and Tg concentration in a finger prick blood sample. The height and weight of each child was also measured. A short questionnaire consisting of socio-demographic questions and an iodine-specific food frequency questionnaire (FFQ) was completed by each child. The study was approved by the University of Otago Health Ethics Committee (Ref# H14/143) and signed consent obtained from children and parents.

A list of New Zealand primary schools was accessed via the Ministry of Education website, Te Kete Ipurangi [14], and used to identify primary schools in Christchurch and in one region of Auckland, which were selected using postcodes in order to reduce travel time for data collection but provide a sufficient number of schools for random selection. Schools from deciles 1–10 were included. School decile is an aggregate measure of the socio-economic status (income, education level and employment) of the school’s community, with schools ranked from decile 1 (the 10% of schools with the highest proportion of students from low socio-economic communities) to decile 10 (the 10% of schools with the lowest proportion of students from low socio-economic communities) [15]. Schools were excluded if the school roll was <100 children or were single-sex. Of 69 eligible schools identified in Auckland, 60 schools were randomly selected using the RAND function in Excel. All eligible primary schools in Christchurch (*n* = 38) were invited to take part as we were advised to expect a low response rate due to continuing disruption in the city following a major earthquake in 2011. The study aimed to recruit 15–20 children from a total of 20 schools for a minimum sample size of 300 children. 

A letter of invitation outlining the study was sent via post to the principal of each school. Schools that agreed to participate were given 60 to 100 information packs, depending on the school roll, to be distributed to classes of children in the target age range. All 8–10 years old children who indicated an interest in the project received an information pack to give to their parent or caregiver, who were asked to complete and return consent forms to the school. The parents of participating children were subsequently notified of the time and date of testing. All data collection took place on school premises between 9:00 a.m. and 17:00 p.m.

### 2.2. Demographic Data and Food Frequency Questionnaire

A questionnaire was administered to each child to obtain information about the child’s date of birth, ethnicity (*i.e.*, New Zealand European and other ethnicities (NZEO), Māori, Pacific, or Asian), use of medications or dietary supplements, general health, followed by a 12-item iodine-specific FFQ. Children were asked about their frequency of consumption of foods over the previous week that are good sources of iodine including milk, dairy products, red meat, chicken, fish, seafood, eggs, iodised salt and fortified bread. Three questions ascertained consumption of a wide range of fortified bread including the number of slices of bread consumed each day, as well as other bread products such as pita bread, bread rolls, bagels, English muffins and sweet buns, and bread-based dishes. As many children were unsure of the type of salt used in the home, children’s responses were clarified by emailing or phoning parents or caregivers. The frequency-of-use categories were as follows: 2 or more times/day; once per day; 5–6 times/week; 2–4 times/week; once/week; less than once/week; or never, and for the question on iodised salt, ‘I don’t know’ could be selected. Information on typical serving sizes other than for sliced bread was not obtained from the children and age-appropriate portion sizes were determined from the New Zealand Food and Nutrition guidelines for children and young people [15]. The iodine content of each food item was calculated using the 2014 New Zealand Food Composition Database (FOODFiles) [16]. The total iodine intake of each food was determined as follows; iodine concentration from food (μg/g) × serving size (g) × frequency factor. If children consumed iodised salt, the quantity of iodine obtained from iodised salt was set at 48 μg/day, equivalent to 1 g of salt/day according to the method used by Edmonds *et al.* [17]. Estimates of iodine intake are presented using two models: a food-only model, and food-plus-iodised salt model.

### 2.3. Urine Collection and Urinary Iodine Analysis 

Spot urine samples were collected by asking children to void urine into a clean plastic bowl placed in a school toilet. An aliquot of urine (10 mL) was transferred to a plastic test tube and kept at 4 °C until samples were shipped to Dunedin where they stored at −20 °C until analysis. UIC was determined using a modification of the method of Pino *et al.* [18] by a single technician at the Department of Human Nutrition, University of Otago. An external reference standard (Seronorm Trace Elements Urine, Sero As, Norway) was analysed with each batch of samples giving a mean iodine concentration of 139 μg/L (published value: 152 μg/L) with a coefficient of variation (CV) of 1.9%. A pooled urine sample was analysed with each batch of samples giving a mean iodine concentration of 97 μg/L and a CV of 5.8%.

### 2.4. Blood Collection and Thyroglobulin Analysis 

A non-fasting fingerprick blood spot sample was collected to determine serum Tg concentration. Each child was asked to select a fingertip that was cleaned with an alcohol swab and allowed to dry. The center of the fingertip was then punctured with a single-use disposable lancet (Tenderlett, International Technidyne Corporation, Edison, NJ, USA) approximately half a centimeter from the nail. The finger was then rotated with the fingertip pointing downward until a blood drop formed, which was lightly touched to filter paper (Whatman 903 Specimen Collection Paper, GE Healthcare Ltd., Auckland, New Zealand) to create a bloodspot approximately one cm in diameter. Filter paper cards were placed in a cardboard box with a lid, so they were not touching each other and not exposed to sunlight, and allowed to dry for 24 h. Filter paper with dried blood spots (DBS) were then placed in a sealed plastic bag, posted to the Department of Human Nutrition at the University of Otago, and stored at −20 °C until analysis. 

For the analysis of DBS Tg, an electrochemiluminescene immunoassay serum Tg assay (Roche Diagnostics New Zealand Limited, Auckland, New Zealand) was adapted for DBS. A 4.8 mm hole punch was used to obtain discs of DBS from filter paper. Blood was extracted by submerging discs in 250 μL of buffer solution (Diluent MultiAssay, Roche Diagnostics New Zealand Limited, Auckland, New Zealand) incubated for 24 h at 5 °C. DBS Tg was measured on an Elecsys 2010. In our laboratory, the correlation between Tg measured in DBS and Tg measured in serum was *r* = 0.91 (*p* ˂ 0.001; *n* = 10). The normal reference range for Tg in children is 4–40 μg/L [19]. Blood samples of children from each school were analysed in the same batch.

The Certified Reference Material Community Bureau of Reference (CRM BCR^®^-457) (Institute for Reference Materials and Measurements (IRMM), Geel, Belgium) was used as an external quality control to check for accuracy of the Tg assay. In our lab, CRM BCR^®^-457 gave a mean of 35.9 ± 1.8 µg/L (expected value: 39.8 µg/L) with a CV of 5.0% (*n* = 12). Pooled serum samples used as an internal quality control gave a mean of 3.3 ± 0.1 µg/L (CV of 3.5%; *n* = 14). Analysis of two levels of Tg controls provided by the manufacturer gave a mean of 22.5 ± 0.6 µg/L (expected value: 15.9–27.7 µg/L) (CV of 2.7%; *n* = 11) and 72.0 ± 2.2 µg/L (expected value: 61.2–106.4 µg/L) (CV of 3.0%; *n* = 11), respectively.

### 2.5. Statistical Analysis 

Microsoft Excel (14.5.0) was used to determine means, medians, percentiles and percentages. STATA (Version 11.0, StataCorp LP, College Station, TX, USA) was used to determine significant predictors of iodine status from the obtained data. Mixed-effect regression analysis adjusting for city and school cluster was used to identify predictors of iodine status. The variables included were age, sex, ethnicity, school decile, and type of salt used (iodised or non-iodised). Pairwise comparisons were used to compare UIC between sex, ethnicity, and school decile. Chi-square analysis was carried out between BMI and age. Because UIC was right-skewed, UIC was log-transformed and geometric means are presented. The level of significance was set at *p* < 0.05 and all tests were two-sided. 

## 3. Results

### 3.1. Recruitment and Sample Size

Eleven out of 60 schools in Auckland and seven out of 38 schools in Christchurch agreed to participate. A total of 1470 recruitment packs were delivered to children aged 8–10 years across the schools and 445 children (30%) consented to take part in the study. Of those who participated, 30 children were excluded. Reasons for exclusion were: having a sibling in the study (*n* = 22); not completing one of either the FFQ, blood sample or urine sample (*n* = 5); outside the age bracket (*n* = 1); thyroid disease (*n* = 1); and withdrawing from the study after sample collection (*n* = 1). The final sample size was 415.

### 3.2. Participant Characteristics

The sociodemographic characteristics of the participants are presented in Table 1 and are compared, when appropriate, to the 2013 New Zealand Census [20]. Most children were aged 9 or 10 years old and there were slightly more females (54%) than males (46%). According to BMI, 72% of children were classified as normal weight, 20% as overweight, and 8% as obese; these values are comparable to data for children aged 2–12 years old from the 2012/2013 New Zealand Health Survey [21]. The majority (56%) of children attended higher decile schools (8–10) indicating higher socioeconomic status.

### 3.3. Urinary Iodine and Thyroglobulin Concentrations

The overall median UIC (25th, 75th percentile) was 116 (82, 156) µg/L (Table 2), which falls between 100 and 199 µg/L indicating adequate iodine status. Furthermore, 5% of participants had a UIC <50 µg/L and 39% had a UIC <100 µg/L, which is below the WHO/UNICEF/IGN criteria for adequate iodine status of 20% <50 µg/L and 50% <100 µg/L, respectively [10]. Using mixed-effect regression analysis there was no significant effect of age (*p* = 0.457), school decile (*p* = 0.705), salt type (*p* = 0.890), and consumption of fortified bread (*p* = 0.329) on UIC. Ethnicity and sex were significant predictors of UIC. Asian children had the highest geometric mean UIC (151 μg/L; median 151 μg/L) followed by Pacific (133 μg/L; median 130 μg/L), NZEO (110 μg/L; median 115 μg/L) and Māori (97 μg/L; median 108 μg/L). There was a significant difference in UIC between Asian and Māori participants (*p* = 0.006) and Asian and NZEO participants (*p* = 0.002). The geometric mean UIC of boys was 133 μg/L (median 131 μg/L), which was significantly higher than the geometric mean UIC of 107 μg/L (median 106 μg/L) in girls (*p* = 0.001). The median Tg concentration was 8.7 µg/L, which falls below 10.0 µg/L indicative of adequate iodine status according to WHO/UNICEF/IGN [10].

Table 3 outlines the contribution the main dietary sources of iodine make to total intake as determined by the FFQ. The mean iodine intake from the food-only model was 65 μg/day. In this model, bread, bread products and bread-based dishes were the largest contributor to iodine intake collectively providing 51% of total daily iodine, with mean intakes of 21 μg, 7 μg and 5 μg of iodine per day, respectively. This was followed by milk and dairy products (28%), eggs (11%), and fish and seafood (6%). The mean iodine intake from food-plus-iodised salt model was 101 μg/day. 

## 4. Discussion

We found that the median UIC in this sample of New Zealand school children was 116 μg/L, falling between 100–199 μg/L indicating iodine sufficiency. The findings of this study are similar to a previous study conducted in 2011 of 8–10 years old children (*n* = 147) in two different New Zealand cities (Dunedin and Wellington) which reported a median UIC of 113 μg/L [12]. However, the present study had a larger sample size, is more representative of the different ethnicities common in the New Zealand population, and the interval between fortification and assessment is longer (*i.e.*, more than five years *vs.* just over one year). Before the mandatory fortification of bread with iodised salt, the median UIC of New Zealand children (*n* = 1153) aged 5–14 years was 68 μg/L [23], which has since doubled. Although UIC is the recommended index to assess iodine status in a population, Tg is proposed to be a more sensitive biomarker of iodine status than other blood indices such as Thyroid Stimulating Hormone (TSH) or thyroxine (T4). The Tg concentration in New Zealand children prior to fortification was 12.9 μg/L [23], which declined to 10.8 μg/L in 2011, and in the current study was 8.7 μg/L. WHO/UNICEF/IGN suggest that a median Tg < 10 μg/L indicates iodine sufficiency [10,24]. To our knowledge New Zealand is the only country that has sequentially measured Tg in children during the transition from iodine deficiency to sufficiency over a 15 year timespan. Together the UIC and Tg data provide confirmation that the iodine status of New Zealand children has improved since the mandatory fortification of bread with iodised salt: New Zealand children now have adequate iodine status.

The higher UIC in males observed in this study is likely a result of boys having higher energy intakes than girls. In the 2002 National Children’s Nutrition Survey (CNS) the median energy intakes differed by 1342 kJ between males and females in the 5–14 years age group [6]. The 2002 CNS also found that the main source of energy in this age group was bread and bread-based dishes (17%). Therefore, not only are boys consuming more energy and thus more likely to have higher iodine intakes, but a higher proportion of their energy is likely to come from fortified bread. Of particular interest was that children of Asian ethnicity had the highest UIC, which has not been noted in previous studies. Little is known about the dietary patterns of Asian children and the group is unlikely to be a homogeneous. However, these children may consume more seaweed, fish and seafood, foods that are rich sources of iodine.

The increase in UIC of school children observed in this study since mandatory fortification of bread is similar to the increase seen in Australian school children [11]. However, the median UIC reported in this study is considerably lower than that reported in 8–10 years old Australian children post-fortification (116 μg/L *vs.* 183 μg/L). One likely explanation for this difference is that pre-fortification the iodine status of New Zealand children was lower than that of Australian children (68 μg/L *vs.* 96 μg/L) [6,8]. Australian children may also consume more bread than their New Zealand counterparts as there is evidence that bread consumption has fallen in New Zealand in recent years [25]. 

The 2002 CNS estimated that only 1% of dietary iodine was supplied from bread, with milk and dairy products making the largest contribution (40%) to iodine intakes in children [6]. FSANZ predicted that bread would supply ~48% of total dietary iodine after the implementation of the strategy [26]. Additionally, a 2012 report by the Ministry of Agriculture and Forestry (MAF) [17] estimated that in children aged 5–14 years old, bread and bread-based dishes would supply 50% of total dietary iodine. The 2011 study by Skeaff and Lonsdale-Cooper [12] found that 47% of the total iodine intakes was from bread and bread-based dishes, while the findings of this study estimated that 51% of dietary iodine was supplied by bread and bread-based dishes when salt was not included. Of note, was the difference between the mean iodine intakes from the food-only and food-plus-iodised salt models (65 μg/day *vs.* 101 μg /day) which suggests that iodised salt is still likely to be a key contributor to iodine in the diet, highlighting the importance of including a measure of iodised salt when assessing dietary iodine intake.

This study had a number of strengths and weaknesses. A key strength of the study was the collection of data from children living in two large cities, which increased the likelihood of obtaining a representative sample of New Zealand children. Indeed, the proportion of children from different ethnicities in this study was comparable to the 2013 Census data. The results of this study combined with the 2011 study provides a sample size of over 500 children [12], providing strong evidence that New Zealand children now have adequate iodine status. One limitation of the study is the low response rate of schools, in particular of low decile schools, which is likely to lead to an under-representation of children from lower socio-economic communities. This may limit the generalisability of the results, although no association between iodine status and socioeconomic status was found in this study. Both this study and the 2011 study by Skeaff and Lonsdale-Cooper [12] assessed children living in urban areas of New Zealand; more than 80% of the New Zealand population lives in an urban area. To our knowledge, the iodine status of New Zealand children living in rural areas has not been investigated, however, one study reported that the energy intakes of rural and urban New Zealand children were not significantly different [27] suggesting similar diets. In this study all the data was collected in March and April (*i.e.*, late summer). Seasonal differences in UIC have been observed in the United Kingdom with higher UIC reported in the winter because dairy cows are given supplemental feeds containing iodine [28]. Such differences in UIC are unlikely in New Zealand because dairy cows are pasture-fed throughout the year. Because of the difficulties associated in quantifying discretionary salt use, caution must be used when interpreting iodine intakes. The FFQ used in this study was not validated although has been used extensively in other published studies of iodine status in New Zealand [17,29,30].

## 5. Conclusions

In conclusion, the results of this study indicate that the iodine status of New Zealand school children has improved since the mandatory fortification of bread with iodised salt. Given the cross-sectional nature of the study, the degree to which mandatory fortification of bread is responsible for this improvement cannot be quantified as other dietary and lifestyle patterns could have changed during this time. It would be useful to further investigate bread consumption in children using a more precise measure of dietary assessment such as diet records, in order to more clearly establish the relationship between fortified bread consumption and iodine intake. We recommend the ongoing monitoring of iodine status in New Zealand children given that dietary patterns can change over time.

## Figures and Tables

**Table 1:** Characteristics of participants in the present study compared with New Zealand (NZ) population data.

Variable		*n* = 415	This Study (%)	NZ Population (%)
Age (Years)	8	55	13	
	9	185	45	NA
	10	175	42	
Gender ^a^	Female	226	54	51
	Male	189	46	49
Ethnicity ^a^	Asian	32	8	12
	Māori	52	13	15
	NZ European	287	69	74
	Pacific	44	11	7
BMI (kg/m^2^) ^d^	Normal	300	72 ^b^	69 ^c^
	Overweight	81	20	21
	Obese	34	8	10
School Decile ^a^	1 to 4	105	25	40
	5 to 7	79	19	30
	8 to 10	231	56	30

^a^ 2013 New Zealand Census [20]; ^b^ Includes 4% of children categorized as underweight.; ^c^ Data obtained from 2 to 12 years old children from 2012/13 Children’s New Zealand Health Survey (*n* = 85,000) [21]; ^d^ BMI calculated using Cole *et al.* [22].

**Table 2:** Urinary iodine concentration (UIC) and thyroglobulin (Tg) of New Zealand school children pre-fortification in year 2002 and post-fortification in years 2011 and 2015.

Index of Iodine Status	2002 ^a^	2011 ^b^	2015 (This Study)	Recommended ^c^
Median UIC (µg/L) ^d^	68 (50, 95)	113 (78, 159)	116 (82, 156)	100–199
% with UIC <50 µg/L	29	12	5	<20
% with UIC <100 µg/L	82	29	38	<50
Thyroglobulin (µg/L) ^e^	12.9	10.8	8.7	<10.0

^a^ Data from 2002 New Zealand Children’s Nutrition Survey of 5–14 years old children (*n* = 1153) [23]; ^b^ Data from 2011 Skeaff and Lonsdale-Cooper study of 8–10 years old children (*n* = 147) [12]; ^c^ WHO/UNICEF/IGN [10]; ^d^ Median (25th, 75th percentiles); ^e^ Median.

**Table 3:** Dietary sources of iodine in sample of New Zealand children using an iodine-specific food frequency questionnaire (FFQ).

Main Contributors to Iodine Intake	Serving Size (g)	Iodine Content (μg/Serve)	Mean (μg/Day)	Median (μg/Day)	% Contribution to Iodine Intake/Day ^a^	
					Pre-Fortification 2002 ^b^	This Study 2015
Milk	250	15.6	14	16	40	28
Dairy	110	7.8	5	3		
Red Meat	100	1.7	<1	<1	7	2
Poultry	100	2	<1	<1		
Fish	100	13.9	2	1	6	6
Seafood	30	34.3	2	0		
Fruit	100	0.5	<1	<1	7	1
Eggs	50	23.1	7	3	8	11
Sliced Bread	26	10.1	21	20	1	51
Other Bread Products	50	20.8	7	3		
Bread-based Dishes	50	26.2	5	4		

^a^ Excludes iodine from iodised salt; ^b^ Data from 2002 New Zealand Children’s Nutrition Survey of 5–14 years old children [17].

## Abbreviations

The following abbreviations are used in this manuscript:

BMI	Body mass index
CNS	Children’s Nutrition Survey
CV	Coefficient of Variation
DBS	Dried blood spot
FFQ	Food Frequency Questionnaire
FSANZ	Food Standards Australia New Zealand
IGN	Iodine Global Network (formerly known as the International Council for the Control of Iodine Deficiency Disorders or ICCIDD)
MAF	Ministry of Agriculture and Forestry
NZ	New Zealand
Tg	Thyroglobulin
TSH	Thyroid Stimulating Hormone
T4	Thyroxine
UIC	Urinary Iodine Concentration
UNICEF	United Nations Children’s Emergency Fund
WHO	World Health Organisation
